# The Liver X Receptor Promotes Immune Homeostasis via Controlled Activation of the Innate Immune System in the Liver

**DOI:** 10.3390/biom15010025

**Published:** 2024-12-28

**Authors:** Hiroyuki Nakashima, Bradley M. Kearney, Manabu Kinoshita

**Affiliations:** Department of Immunology and Microbiology, National Defense Medical College, Saitama 359-8513, Japan; bmkearney@ndmc.ac.jp (B.M.K.); manabu@ndmc.ac.jp (M.K.)

**Keywords:** liver X receptor, Kupffer cells, apoptosis, cholesterol, hepatocyte

## Abstract

The liver is an indispensable metabolic organ, responsible for accumulating and transporting various nutritional compounds in hepatocytes. However, the transport of these materials from the liver is an energetically intensive task because they contain a considerable number of hydrophobic components, including free cholesterol, and require specialized transfer proteins to shuttle these substances through an aqueous phase. Liver X receptors (LXRs) induce the expression of cholesterol transporters in macrophages to transport free cholesterol derived from apoptotic cells into extracellular space via high-density lipoproteins. Additionally, LXRs control innate immune cells through two major mechanisms: upregulating the phagocytic activity of macrophages and suppressing inflammatory reactions to prevent aggressive activation of immune cells. Therefore, the primary role of LXRs is to accelerate efferocytosis without provoking inflammation and facilitate the transfer of free cholesterol from the intracellular space. This mechanism makes the innate immune system a substantial contributor to systemic metabolic control. Concomitantly, LXRs are important factors in regulating systemic defense mechanisms through the efficient regulation of immune cells. LXR activation, therefore, has great potential for clinical applications in the treatment of metabolic, infectious, and autoimmune diseases. In this review, we discuss the current understanding of the link between LXRs and innate immune cells in the liver, along with prospects for clinical applications of LXR agonists.

## 1. Introduction

Proper maintenance of both metabolic and immune function is a key factor in healthy, complex organisms. Although these two completely different biological functions seem at first to function independently, they are, in fact, closely linked and regulate each other. The liver is the largest and most important metabolic organ in the human body. It processes various nutrients absorbed from the gastrointestinal tract, excretes numerous essential proteins, stores energy as glycogen and lipids, and transports excessive cholesterol to produce bile. Additionally, it receives abundant blood flow from both systemic and portal circulations, forming a vast amount of microcirculation called the sinusoid. Within the sinusoidal microcirculation of the liver, there exists a defense network of innate immune cells, such as Kupffer cells, Natural Killer (NK) cells, and Natural Killer T (NKT) cells [1,2]. This unique environment makes the liver a key focus area for immunology research [3]. Not surprisingly, the liver is where metabolism and immune function intersect, making it an ideal subject for studying the various mechanisms that link these essential functions together. According to previous reports, nutrients such as free cholesterol significantly influence the function of innate immune cells in the liver and can cause metabolic dysfunction-associated steatotic liver diseases when the balance is disrupted [4]. The nuclear receptor “Liver X receptor (LXR)” plays a crucial role in the transport of free cholesterol from hepatocytes and liver resident macrophages [5], and its intracellular content is a key factor in determining immune cell functions [6]. Therefore, LXR is the crucial mechanism connecting metabolism and immune function and is an attractive target for studies to elucidate the etiology of metabolic dysfunction-related diseases.

## 2. LXRs Are Key Nuclear Factors That Link Metabolism and Immunity

The LXR family of nuclear receptors regulates the expression of proteins involved in lipid and cholesterol metabolism [5]. The endogenous ligands of the LXR family are hydrophobic cholesterol-related metabolites, such as oxysterols [7,8], and precursors of cholesterol biosynthesis, such as desmosterol [9]. Intracellular accumulation of these ligands activates LXRs, which induces expression of the cholesterol-related transporter ABC-binding cassette transporter, A1 (ABCA1), promotes the excretion of excess cholesterol into the extracellular space via high-density lipoproteins (HDLs) [10,11,12]. Additionally, LXR signaling induces the expression of apolipoprotein E (APO-E), a key mediator in cholesterol excretion [13]. In addition to cellular efflux, LXRs facilitate the movement of excess cholesterol from the periphery into the gastrointestinal tract via bile in a process known as reverse cholesterol transport (RCT) via ABCG1 [14,15]. Furthermore, LXR decreases cholesterol uptake into the intracellular space via LDL-R [16]. Based on these findings, LXR has been recognized as a sensor of excessive cholesterol that induces cellular cholesterol efflux and RCT [17].

**Figure 1:**
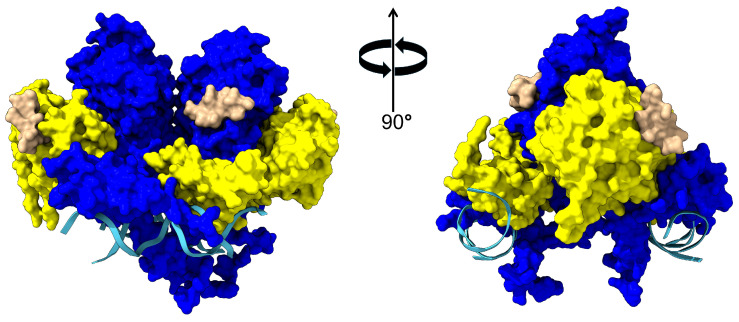
Complex between LXR, RXR, and DNA. The crystal structure of the complex (PDB code 4NQA) is shown with the LXR in blue, RXR in yellow, DNA in cyan, and co-repressors in beige. Ligand binding by LXR induces a conformational change that results in the dissociation of the co-repressor elements and subsequent recruitment of transcriptional activators such as CREB binding protein and p300 [23].

There are two isoforms of LXR, LXRα and LXRβ, that form heterodimers with their respective obligate partner retinoid X receptors (LXRα/RXRβ and LXRβ/RXRα) [18]. The two LXR isoforms are encoded by different genes, are primarily alpha-helical in structure, and contain a ligand binding domain and a DNA binding domain [19]. The ligand binding domain isoforms share approximately 75% amino acid identity, and X-ray crystallography studies reveal a high degree of structural homology with an overall RMSD of 1.17Å [20]. The amino acids comprising the ligand binding domain are hydrophobic and thus it has a higher affinity for hydrophobic ligands. When the LXR/RXR heterodimer is not bound to an agonist, the heterodimer binds to DNA, but also recruits the co-repressors N-CoR (nuclear receptor co-repressor) and SMRT (silent mediator of retinoic acid receptor and thyroid receptor) (Figure 1) [21]. While the LXR/RXR heterodimer is bound to these co-repressors, transcription of the proteins associated with cholesterol transport mentioned above is repressed. However, the binding of LXR or RXR agonists induces structural changes in the heterodimer that release the co-repressors, which subsequently allows for the transcription and translation of LXR-associated proteins [22].

The two isoforms of LXR are also differentially expressed in the various tissues of the body with LXRα expressed specifically in the liver, gastrointestinal tract, adipose tissue, and kidneys, while LXRβ is ubiquitously expressed in all tissues (Table 1) [24]. These differences extend not only to organ-specific parenchymal cells but also to immune cells where myeloid and B1 B cells express LXRα [25]. LXRα-deficient mice exhibit similar liver structure and functions as normal mice. However, when fed a high-cholesterol diet, free cholesterol immediately accumulates in hepatocytes in these mice [26]. Therefore, one potential mechanism of dietary cholesterol intolerance involves LXRα deficiency, which results in the accumulation of free cholesterol in the intracellular spaces of hepatocytes due to disruption of the cholesterol excreting pathway. On the other hand, in LXRβ deficient mice, there is no significant difference in liver cholesterol content even after a high-cholesterol diet [27]. These phenomena strongly suggest that LXRα, rather than LXRβ, is the primary factor involved in cholesterol excretion. LXRα/β double knockout mice exhibit severe inflammatory reactions in the liver by inducing strong activation of macrophages [28] and stellate cells [29], indicating that the combination of LXRα/β has a substantial inhibitory effect on immune reactions.

## 3. LXRs Are Crucial for Maintaining the Functions of Macrophages

An interesting phenomenon of LXR signaling is its secondary effects on immune mechanisms such as phagocytosis and the bactericidal activity of macrophages. Intracellular cholesterol metabolism is closely associated with the phagocytic activity of macrophages against bacteria. The activation of TLR3/4 by external pathogens inhibits LXR-dependent cholesterol efflux and induces intracellular cholesterol accumulation within macrophages [33]. This interaction is mediated by IRF3, a specific effector of TLR3/4, which inhibits the transcriptional activity of LXR. Moreover, mice lacking LXRα are susceptible to infection by intracellular organisms, such as *Listeria monocytogenes*, due to increased susceptibility to apoptosis in macrophages during the immune response [30]. Consistent with this finding, another study showed that LXR activation induces anti-apoptotic proteins and protects macrophages from apoptosis to maintain their continued function during infection [34]. In addition to *Listeria*, LXRα is involved in the bactericidal activity of other intracellular bacteria such as *Mycobacterium tuberculosis* [31] and *Salmonella typhimurium* [32]. LXR activation counteracts bacteria-induced macrophage dysfunction in an NAD+/CD38-dependent manner, thereby restoring cytoskeleton integrity and preventing intracellular replication of pathogenic bacteria [32]. Together these results demonstrate that LXRs are key contributors to innate immune reactions against pathogenic bacteria with potent virulent activity.

## 4. LXRs Are Essential for the Development and Maintenance of Liver Kupffer Cells

### 4.1. Kupffer Cells Are a Special Class of Liver-Resident Macrophages

**Figure 2:**
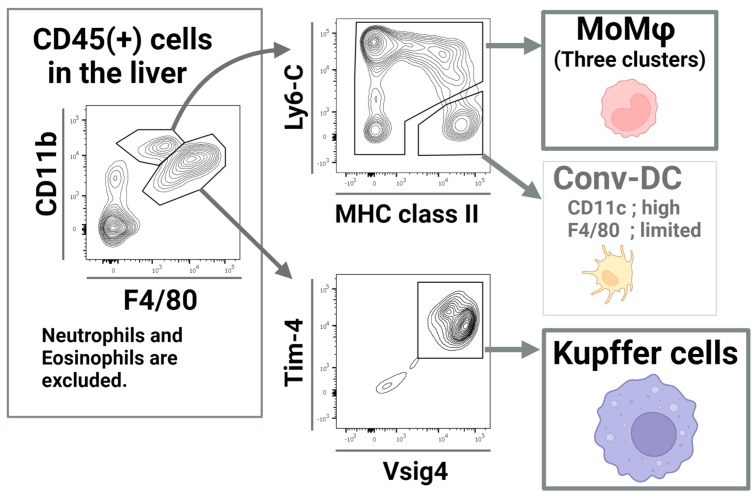
Two major F4/80 positive populations of immune cells exist in the liver [38]. The F4/80 low population contains three subclusters of monocyte-derived cells as well as dendritic cells, each exhibiting varying levels of F4/80 expression, from limited to medium in each subcluster. Kupffer cells showed substantially high expression of F4/80, Vsig4, and Tim-4. (Created with BioRender.com).

Kupffer cells are among the most well-known and well-studied macrophages. In 1876, Carl von Kupffer first discovered non-parenchymal cells that exhibited endocytic capacity within the liver sinusoid [35,36]. Since then, these cells have remained a perennial research topic in macrophage research [37]. This cell population is a canonical example of a tissue-resident macrophage population that tightly adheres to organ tissue and exerts organ-specific function. The primary function of Kupffer cells is to eliminate circulating bacteria via phagocytosis, which substantially contributes to systemic defense. Several studies have examined their functions, origins, and relationships with metabolic functions. Technological advances over the years such as single-cell, fate-map, and flow-cytometric analysis, have uncovered both the developmental origins and functional differences in Kupffer cells (Figure 2).

### 4.2. LXRα Subunit Is Involved in the Normal Development of Kupffer Cells

Self-renewal is an important pathway for maintaining the number of macrophages. Kupffer cells, Langerhans cells in the skin, and microglia in the brain all originate from yolk sac progenitor cells and are self-maintained in adult tissues [39]. In the development process, transcription factor ZEB2 plays a crucial role in the self-renewal and acquisition of Kupffer cell identity in an LXRα-dependent manner [40]. Besides self-renewal, Kupffer cells can be replenished by blood monocytes, which can differentiate into fully functional cells in the sinusoidal microenvironment [41]. In this monocyte-origin development, LXRα also plays a significant role [42]. Therefore, LXRα is a vital factor in the development of Kupffer cells both from Yolk-sac progenitor cells and from bone marrow-derived monocytes. However, in LXRα-deficient mice, the number of Kupffer cells is reportedly the same as in normal mice on a normal diet [43]. This result suggests that some other factors may compensate for the function of LXRα in the developmental process. Although their number is intact, LXRα-deficient mice show severe dietary cholesterol intolerance [26], and Kupffer cells are severely activated after a high-cholesterol diet [43]. This result suggests that Kupffer cells in LXRα-deficient mice have a significant characteristic, which is that they are easily converted to a pro-inflammatory state after dietary cholesterol intake.

### 4.3. LXRs Accelerate Homeostatic Metabolic Process by Activating Kupffer Cells

In the spleen, certain macrophage subpopulations are actively involved in heme and iron metabolism. Those macrophages located in the red pulp have large and complex structures and their primary function is the phagocytosis of senescent red blood cells [44]. During the breakdown of phagocytosed red blood cells, macrophages accumulate a significant amount of iron released from heme. Iron transport, therefore, is an important step in this process. Notably, in vitro studies show that LXRα is also closely involved in facilitating iron efflux from macrophages via the Nrf2-dependent pathway [45]. Although the functional link between LXRα and splenic red pulp macrophages requires further elucidation, it is possible that iron transportation is regulated by LXR [46]. Similar to that in the spleen, a relationship between iron metabolism and macrophages in the liver has been reported. In 2001, Hirano et al. reported that Kupffer cells actively engulf circulating denatured red blood cells and participate in the transport of the heme derived from these cells to generate bilirubin [47]. This finding is significant because it highlights the primary role of immune cells in physiological metabolic processes. In 2010, Graham et al. reported that hepatic iron content correlated with intrahepatic cholesterol content, whereas no relationship with serum cholesterol concentrations was observed [48]. This suggests that iron metabolism in the liver is closely related to hepatic cholesterol levels. These findings indicate that Kupffer cells are active players in homeostatic metabolism, including the transport of cholesterol, heme, iron, and bilirubin. Considering that splenic macrophages accelerate heme transport via LXR signaling, LXR may similarly accelerate the transport of these substances in the liver via Kupffer cell activation.

### 4.4. LXRs Augment the Phagocytosis of Apoptotic Cells by Kupffer Cells

**Figure 3:**
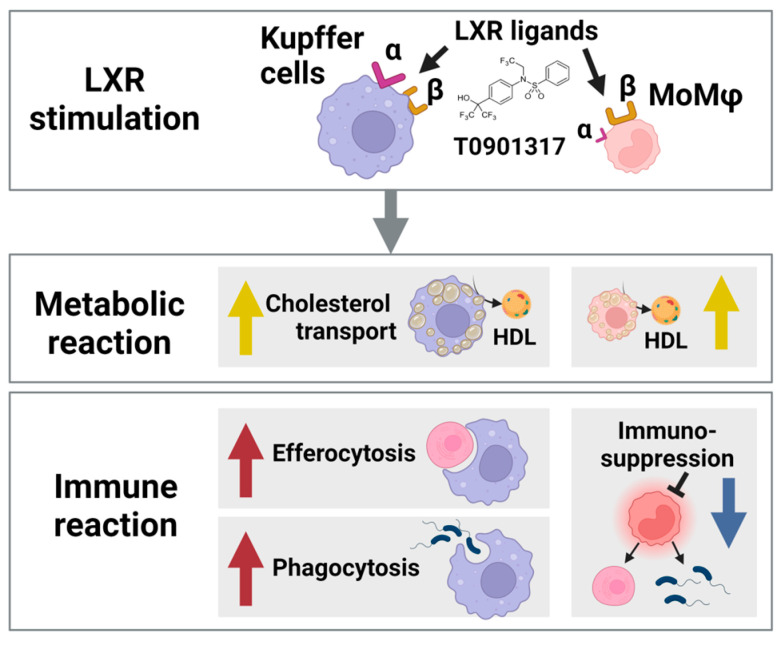
LXR stimulates Kupffer cells in three ways: increasing cholesterol transport to lipoproteins, promoting efferocytosis of apoptotic liver cells, and enhancing phagocytosis of invasive bacteria. LXR induces an immunosuppressive effect in Monocyte-derived macrophages (MoMφ), polarizing them toward tissue remodeling and repair. (Created with BioRender.com).

One of the essential functions of macrophages is the elimination of damaged or apoptotic cells from tissues through phagocytosis (Figure 3). This function, known as efferocytosis, is a crucial mechanism for maintaining homeostasis in the body. Apoptotic cells contain substantial amounts of cholesterol and related chemical structures that need to be eliminated from the body. LXR and its downstream transporters play crucial roles in this process by facilitating cholesterol efflux and transporting intracellular cholesterol into HDL from the intracellular space. LXR stimulation upregulates MerTK expression in macrophages, which function as sensors for apoptotic cells [49]. Apoptotic cell engulfment by macrophages also induces LXR-independent ABCA1 expression, which is essential for efflux of free cholesterol [50]. In addition to cholesterol, hydrophobic chemicals from engulfed apoptotic cells are transported to the extracellular space by various transporters [51]. The liver is a highly regenerative organ where hepatocytes continuously undergo self-renewal through the process of mitosis and apoptosis. Therefore, Kupffer cells must rapidly and efficiently eliminate apoptotic hepatocytes to maintain liver homeostasis. To facilitate this, Kupffer cells express high amounts of the sensor protein for apoptotic cells called MerTK [52,53]. Considering the relationship between LXRs and MerTK expression, LXR stimulation of Kupffer cells is essential for eliminating apoptotic hepatocytes containing high levels of cholesterol and fatty acids. The relationship between LXRs and apoptosis sensor proteins on Kupffer cells is an attractive research topic.

### 4.5. LXRs Augment the Phagocytosis of Bacteria of Kupffer Cells

The primary function of Kupffer cells is to eliminate bloodborne bacteria via phagocytosis. These cells constantly engulf pathogenic bacteria present in both systemic and portal circulation. Their phagocytic activity is so robust that they can begin to capture intravenously injected bacteria within a few minutes [54]. The clearance kinetics of gram-positive bacteria are notably faster than those of Gram-negative bacteria [55], primarily because of the presence of a specific receptor molecule, CRIg, on Kupffer cells. This receptor facilitates the recognition of components of Gram-positive bacteria [56] and does not require opsonization or a complement system. These findings show that Kupffer cells are specialized macrophages that target bacteria circulating in the blood. Abrogation of both LXRα/β reduced in vivo phagocytosis of *Escherichia coli*, while LXR stimulation upregulated in vitro phagocytosis [53]. This finding demonstrates the involvement of LXR in bacterial phagocytosis by Kupffer cells. The mechanism underlying the bactericidal functions of macrophages, such as peritoneal [33] and bone marrow-derived macrophages [30,32,34] have also been elucidated. However, the mechanisms specific to Kupffer cells are still not well understood owing to the challenges associated with isolating this cell population from the liver to assess their biological functions in vitro.

### 4.6. LXRs and Subpopulations of Kupffer Cells

Recent single-cell RNA sequencing technologies revealed that two different populations exist in embryo-derived Kupffer cells, the CD206^lo^ ESAM^-^ population (KC1) and the CD206^hi^ ESAM^+^ population (KC2) [57,58]. KC1 is the major population comprising around 80% of total Kupffer cells, and the minor population KC2 shares antigens with sinusoidal endothelial cells such as CD31. Although KC2 is a relatively minor population, it is associated with metabolic diseases by stimulating fat storage in the liver and exacerbates steatohepatitis after HFD consumption via CD36 [58]. In this report, the authors show that LXRα gene Nr1h3 is expressed in both KC1 and KC2. However, there is no difference in expression levels between these two populations. These LXR gene expression results were obtained from steady-state normal mouse liver, and more studies are needed to elucidate the LXR functions in KC2 after HFD consumption. Other Kupffer cell populational differences were reported based on three different Kupffer cell markers, Vsig4, Tim-4, and CD163, in the experimental steatohepatitis model by an independent researcher [59]. In the experimental steatohepatitis model, monocyte-derived Tim-4(-) Kupffer cells (MoKCs) increase in number and replace embryo-derived Tim-4(+) Kupffer cells (EmKCs). These MoKCs have an inflammatory nature and play a key role in aggravating steatohepatitis. MoKCs are monocyte-derived cells and are different from embryo-derived KC2 which is defined by Bleriot. On the other hand, the authors also show the existence of two different populations in EmKCs in a normal state based on CD163 expression. Following this report, CD163(+) Kupffer cells in normal diet mice are reported to have higher bactericidal activity compared to CD163(-) cells [38]. The analysis of subpopulations in Kupffer cells is still under intensive investigation by many researchers, and future research and discussion will reveal the relationship between each LXR receptor and populational subclass.

## 5. LXRs Induce Immuno-Regulatory Reactions

Stimulation of LXRs induces anti-inflammatory and immune-regulatory functions under various conditions [60]. This mechanism is an attractive area of research because of its potential clinical application to use LXR stimulants to treat atherosclerosis and other metabolic disorders where chronic inflammation underlies their pathogenesis. Additionally, LXR stimulants may potentially be used to treat autoimmune diseases because the endogenous LXR ligand oxysterol has been shown to play a substantial role in regulating autoimmune reactions [61].

### 5.1. LXRs Suppress Inflammatory Signals in Immune Cells

In 2003, Joseph et al. reported that stimulation of LXRs suppressed the expression of inflammatory signals, such as inducible nitric oxide synthetase, cyclooxygenase-2, and IL-6 [62]. Additional studies have identified several crucial mechanisms of immune suppression involving LXR. The induction of anti-inflammatory metalloproteinase 9 (MMP-9) [63] and arginase II [64] suggests that LXR activation stimulates various macrophage populations to undergo M2 polarization. Moreover, the LXR-induced cholesterol transporter ABCA1 regulates inflammatory reactions by inhibiting TLR signaling [65] and upregulating IL-10 expression [66].

### 5.2. LXRs Augment the Production of Immune-Regulatory Fatty Acids

The inflammatory reaction is tightly regulated by a variety of co-repressors in the nuclei of macrophages. Among these, N-CoR represses LXR expression in macrophages. The derepression of LXR in an inducible knockout of N-CoR results in an immunoregulatory state that alleviates glucose intolerance despite fat accumulation in the liver [67]. Activation of LXR accompanies increased levels of ω3 unsaturated fatty acids, such as eicosapentaenoic acid (EPA) and docosahexaenoic acid (DHA). Polyunsaturated fatty acids (PUFA), such as EPA and DHA, exhibit anti-inflammatory effects and their accumulation in the liver and fat tissues improves chronic inflammation and ameliorates glucose tolerance. LXR activation not only enhances PUFA production but also increases the incorporation of PUFA into phospholipids through the activation of the Lpcat3 enzyme, thereby improving cellular membrane function and reducing endoplasmic reticulum stress induced by saturated fatty acids [68].

## 6. LXR Stimulation Reduces Inflammatory Response Induced by Liver Monocyte-Derived Macrophages (MoMφs)

### 6.1. Liver MoMφs Are Distinct Macrophages Derived from Monocytes

The F4/80 low and CD11b high macrophage populations in the liver are distinct from Kupffer cells and originate from circulating blood monocytes (Figure 2). They also differ from blood monocytes in their high expression of the macrophage marker CD64 and low expression of the monocyte marker CD115 [38]. These cells are actively recruited to the liver, where they perform liver-specific functions within the sinusoidal space. The function of liver MoMφs is to augment the overall immune response by producing cytokines that promote localized inflammation in the liver [69]. In 2014, Sato et al. reported that this specific cell population is a crucial effector in various types of experimental hepatitis, e.g., carbon tetrachloride-induced acute hepatitis [70]. Notably, a high fat and cholesterol diet increases the number and cytokine-producing capacity of these macrophages, which in turn augments liver injury in α-galactosylceramide and common bacterial DNA-induced hepatic injury [71]. These findings suggest that this population plays an essential role in the inflammatory response in the liver. Interestingly, in a recent study, F4/80 low MoMφs were found to contain at least four different cell clusters [38]. Specifically, MHC class II positive pro-inflammatory MoMφs are implicated in the inflammatory response under various experimental models. These findings are consistent with studies on blood monocyte subsets obtained using single-cell analysis [72,73]. Re-evaluation of the mechanism of experimental hepatitis using advanced cluster analysis of liver MoMφs is an attractive research target to deepen our understanding of liver immune mechanisms and increase the granularity in the litany of roles played by specific subtypes of immune cells.

### 6.2. LXRs Regulate MoMφs to Avoid Excessively Aggressive Immunological Responses

In LXR double knockout mice, the number of MoMφs in the liver increased as compared to control mice, which resulted in a severe inflammatory response following various stimuli even under normal dietary conditions [28]. Consequently, the combination of LXR α and β subunit is indispensable for the regulation of the recruitment of MoMφs from the bone marrow and suppresses the aggressive inflammatory response (Figure 3), which is consistent with the concept from previous studies [60]. In LXRα deficient mice, the number and function of MoMφs are similar to the control mice. However, when these mice consume a high-cholesterol diet, they quickly develop severe steatosis in the liver, and the number and functions of liver MoMφs significantly increase, showing an aggressive response following LPS treatment [43]. This observation suggests that LXRα is closely related to cholesterol metabolism and actively involved in the regulation of MoMφs in dietary cholesterol overload. The relationship between LXR stimulation and the composition of sub-classifications of MoMφs is an additional attractive research topic.

## 7. Advancements in Clinical Applications of LXR Activation Therapy

LXR activation prevents severe inflammatory reactions during phagocytosis and pathogen elimination, presenting an attractive therapeutic strategy for various diseases, especially metabolic diseases where chronic inflammation occurs in adipose tissue, atherosclerotic plaques, and the liver. The most attractive field of clinical applications of LXR stimulation is the treatment of metabolic dysfunction-associated steatohepatitis (MASH) [74]. This application is not limited to just metabolic steatohepatitis but also other liver diseases such as alcoholic liver diseases [75], viral hepatitis [76,77], and hepatocellular carcinoma [78]. The three features of LXR activation (accelerating cholesterol transporters, suppressing inflammatory reactions, and augmenting apoptotic cell clearance) are all attractive targets for a variety of metabolic diseases.

### 7.1. Stimulation of LXRs Promotes Lipid Accumulation in Liver and Adipose Tissue

**Figure 4:**
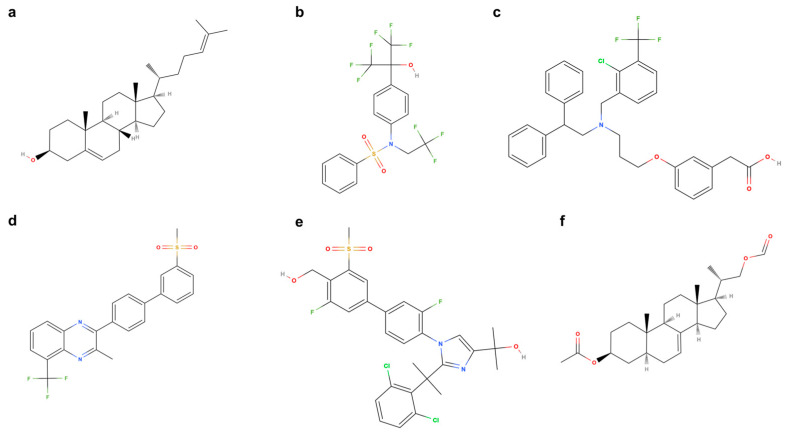
Chemical structures of endogenous ligands and drugs that bind to LXRs (**a**) Demesterol (**b**) T0901317 (**c**) GW3965 (**d**) WYE-672 (**e**) BMS-852927, and Δ24-dehydrocholesterol reductase inhibitor (**f**) SH42.

Although the endogenous ligand desmosterol (Figure 4a) is known to stimulate the LXR pathway, synthetic drug development is the gold standard for producing a desired effect while minimizing off-target effects. In 2000, the first-generation synthetic LXR agonist, T0901317 (Figure 4b), was reported to activate lipogenesis via the LXR/SREBP-1 axis [79]. This compound substantially improved glucose tolerance in vivo [80], and its clinical use has been highlighted since its publication [81,82]. However, undesirable side effects, such as severe hepatic steatosis, hypertriglyceridemia, and off-target stimulation of the farnesoid X receptor (FXR) [83] and pregnane X receptor (PXR) [84], have greatly tempered the prospect of this compound’s general clinical use. The specific LXR α/β agonist, GW3965 (Figure 4c), was developed to mitigate these off-target effects and [84,85] demonstrated substantial upregulation of reverse cholesterol transport in hyperlipidemic mice [86]. Due to its high degree of LXR specificity, this compound has been frequently used in animal experiments and human clinical trials are currently ongoing [87]. However, LXR stimulation using synthetic ligands inevitably results in a synergistic activation of the lipogenic cycle. As a result, fat accumulation in the liver and adipose tissue is a persistent side effect of this therapy in its current form [88]. This serious side effect undermines the prospects of LXR activation therapy for some metabolic diseases, especially MASH. Several strategies have been proposed to overcome these adverse effects to varying degrees of success.

### 7.2. Selective Stimulation of the LXRβ Subunit Is Insufficient to Prevent Excessive Fat Accumulation

For the development of fatty accumulation in the liver and adipose tissue, LXRα in the liver plays a more significant role than the β subset [89,90]. Based on this finding, selective LXRβ-specific agonists, such as WYE-672 (Figure 4d) [91,92] or BMS-852927 (Figure 4e) [93,94], were developed for clinical use. Although various clinical and pre-clinical trials in human and experimental animals revealed that even selective activation of LXRβ subclasses accompanies lipogenic adverse effects [95], the activation of SREBP-1c is less than in previous compounds [96]. Moreover, as previously mentioned, hepatic LXRα primarily regulates inflammation and cholesterol excretion, suggesting that selective LXRβ antagonists may offer limited therapeutic benefit. Therefore, a more efficient therapeutic strategy, using a different approach, is required for future applications.

### 7.3. Nanoparticle Drug-Delivery Systems Can Overcome the Serious Off-Target Effects of LXR Stimulation

**Figure 5:**
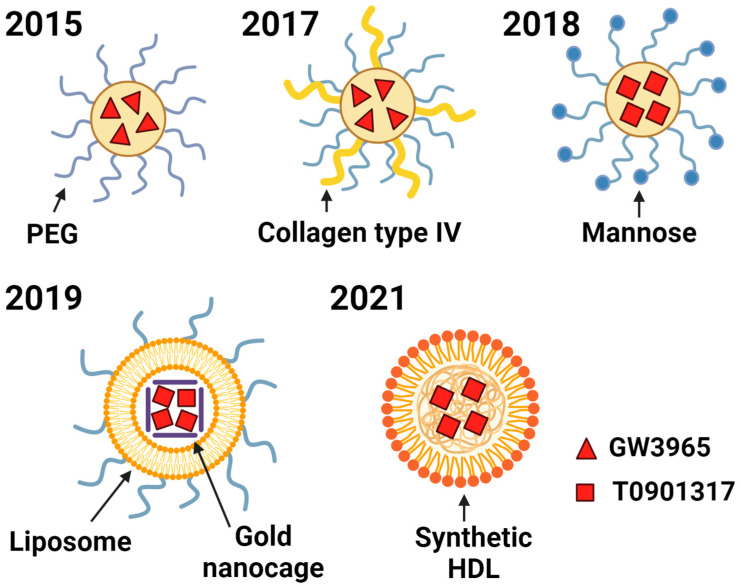
Recent advances in nanoparticle encapsulated LXR ligands. All LXR ligands are hydrophobic and do not remain soluble in body fluids, limiting their ability to reach pathogenic sites. Nanoparticle-encapsulated substances with PEGylated strands were first developed to overcome this challenge in 2015 [97]. Modified particles have been further developed that contain type IV collagen to target atherosclerotic sites [98] and mannose for enhanced uptake by macrophages [99]. Liposome-encapsulated gold nanocage [100], and synthetic lipoproteins [101] have also been developed for the effective delivery of LXR ligands. (Created with BioRender.com).

One of the major problems with LXR agonists is their hydrophobic nature, which hinders their solubility in body fluids and limits their ability to reach pathogenic sites. Increasing administrative doses to enhance access to these pathogenic organs, may be accompanied by non-specific distribution to non-pathogenic organs, thereby increasing the risk of unwanted side effects. To address this issue, nanoparticles containing LXR ligands were developed, and substantially improved results were reported to increase solubility in water, with substantial ameliorative effects on atherosclerotic lesions observed without inducing hepatic steatosis [97] (Figure 5). Subsequently, the modification of nanoparticles by adding a collagen type IV targeting material increased the efficiency of the delivery of the particles to the atherosclerotic plaques [98].

Following the successful reports of nanoparticle-encapsulated LXR agonists, various types of particles have been developed and are still under progressive research. Notably, the first-generation LXR activator T0901317 does not induce lipogenic side effects when coated with nanoparticles, such as mannose-functionalized dendrimeric nanoparticles [99], liposome-coated gold nanocages [100], and synthetic HDL nanoparticles [101]. This is crucial because the ligands administered in the nanoparticle-incorporated form do not stimulate SREBP pathways. This is believed to occur through macrophages. The nanoparticles are not directly absorbed by hepatocytes or adipose cells but are incorporated by macrophages through phagocytosis. Within macrophages, the ligands are exposed in the intracellular space, where they either directly exert their effect or are incorporated into the transport pathway of hydrophobic materials. This approach highlights LXR agonist therapy, especially for liver diseases, such as MASH.

Additionally, the modification of nanoparticles with phosphatidylserine triggers uptake and phagocytosis via the apoptosis cell receptor, MerTK or Tim-4 [102]. As previously mentioned, Kupffer cells express high levels of these apoptotic cell detection markers, making them excellent targets for delivery via nanoparticles with mocked “eat-me” signals. Efficient delivery of the LXR ligand to Kupffer cells could enhance LXR stimulation therapy for MASH, offering an effective therapeutic strategy. The development of applicable nanoparticles via oral medication or percutaneous injection is necessary for safe and practical administration.

### 7.4. LXR Stimulation with Endogenous Metabolites Is an Attractive Solution for Clinical Applications

After administration from the outside, synthetic ligands must pass through specific anatomic spaces to reach the pathological site and need to encounter other metabolic receptors during this process, which can induce unexpected reactions. To overcome this side effect, increasing internal endogenous LXR ligands is another attractive strategy for effectively activating LXRs because it can exert its potential naturally on the proper metabolic chain without stimulating other pathways. Several types of endogenous materials have been reported as ligands for LXR, and intermediate metabolites in cholesterol biosynthesis are strong activators of LXRs. Among these, desmosterol [103], which is the final intermediate substrate of cholesterol biosynthesis, effectively stimulates LXR and reduces inflammatory response genes and macrophage foam cell transformation in hypercholesterolemic mice [9]. Desmosterol induction is a novel target of the LXR activation strategy because it does not upregulate SREBP [9,104].

Desmosterol is the immediate precursor to cholesterol in the Bloch pathway and is directly converted to cholesterol via the enzyme Δ24-dehydrocholesterol reductase (DHCR24). Accordingly, inhibition of DHCR24 results in the accumulation of desmosterol, which is an attractive strategy for LXR stimulation using its endogenous ligand [105]. After extensive studies to identify a highly selective and safe DHCR24 inhibitor, SH42 (Figure 4f) was found to be the most effective candidate [106]. This compound effectively blocked DHCR24 and led to the subsequent accumulation of desmosterol resulting in stimulation of LXR. As a result, LXR induced the expression of cholesterol-specific transporters and the production of PUFA in vivo. This in turn ameliorated murine peritonitis [107]. In a follow-up study, Zhou et al. tested the effectiveness of SH42 on the treatment of an experimental steatohepatitis model where mice that were administered SH42 showed amelioration of inflammatory reactions and fibrosis [108]. In this model, indirect targeting of LXR activation as a preventive therapy not only decreased fat accumulation in hepatocytes but also exhibited an immunosuppressive treatment effect. Thus, SH42 is an ideal drug for MASH treatment because of its preventive effects on steatosis and inflammation.

Consistent with the highly hydrophobic character of the ligand binding domain, many compounds targeting LXR have a high degree of hydrophobicity. Indeed, most of the drugs discussed in this review, including SH42, violate one of Lipinski’s rule of five [109] due to an octanol-water partition coefficient greater than 5. This makes drug optimization increasingly difficult without triggering additional violations of the rule of five, which would limit clinical applicability. However, using strategies that combine LXR-targeting drugs with nanoparticle drug delivery platforms could potentially facilitate the development of efficient drugs for the treatment of MASH. Orally administered nanoparticles tend to accumulate in the liver. Therefore, the combination of SH42 and orally administered nanoparticles coated with signal molecules to induce uptake and phagocytosis by Kupffer cells is an attractive strategy to reduce off-target effects and overcome physicochemical drug limitations to develop effective MASH therapies.

## 8. Conclusions

LXR is a key regulating factor that connects metabolic and immune functions and is deeply involved in defense against pathogens and maintaining physiological homeostasis. By studying this protein and its effects, researchers have been able to elucidate the molecular underpinnings of immune mechanisms. Additionally, LXR is a promising drug target for treating diseases that involve metabolic and inflammatory mechanisms. Although the development of LXR-stimulating drugs has been slow due to off-target effects and the difficulty of developing a hydrophobic drug that exhibits favorable ADME traits, recent breakthroughs in drug delivery have shown great promise in overcoming these limitations. We believe that LXR will continue to be an attractive research subject from basic and clinical medicine perspectives in the years to come.

## Figures and Tables

**Table 1:** The relations between organs, immune cell types, and functions of each LXR subset are summarized [24,25,26,27,28,29]. LXRα is more restricted to specific organs and immune cells involved in fat storage, cholesterol transport, and excretion. LXRα is deeply involved in the excretion of excess cholesterol in the liver [26], whereas LXRβ is not [27]. As for immune suppression, synergistic activation of both subsets is necessary in normal diet [28]. ++; deeply involved, +; involved, -; not involved.

	Organs and Functions	LXRα	LXRβ
Systemic functions	Organs in which each LXR is expressed [24]	Liver Gastrointestinal tract Adipose tissue Kidney	All organs
	Excretion of excess cholesterol into bile [26,27]	++	-
Immune functions	Cell types [25,28] in which each LXR is expressed	Resident macrophages B1 cells	All Immune cells
	Cholesterol transport to HDL [26,27]	++	+
	Immuno-suppression [28,29]	+	+
	Phagocytosis and Bacterial killing [30,31,32]	+	-

## Data Availability

Not applicable.
